# Early-stage idiopathic Parkinson’s disease is associated with reduced circular RNA expression

**DOI:** 10.1038/s41531-024-00636-y

**Published:** 2024-01-20

**Authors:** Benjamin J. Whittle, Osagie G. Izuogu, Hannah Lowes, Dasha Deen, Angela Pyle, Jon Coxhead, Rachael A. Lawson, Alison J. Yarnall, Michael S. Jackson, Mauro Santibanez-Koref, Gavin Hudson

**Affiliations:** 1 Wellcome Centre for Mitochondrial Research, Biosciences Institute, Newcastle University, Newcastle upon Tyne, UK; 2 European Molecular Biology Laboratory, European Bioinformatics Institute, Wellcome Genome Campus, Hinxton, Cambridgeshire UK; 3 Wellcome Centre for Mitochondrial Research, Translational and Clinical Research Institute, Newcastle University, Newcastle upon Tyne, UK; 4 Biosciences Institute, Newcastle University, Newcastle upon Tyne, UK; 5 Translational and Clinical Research Institute, Newcastle University, Newcastle upon Tyne, UK

**Keywords:** Gene regulatory networks, Preclinical research, Predictive markers

## Abstract

Neurodegeneration in Parkinson’s disease (PD) precedes diagnosis by years. Early neurodegeneration may be reflected in RNA levels and measurable as a biomarker. Here, we present the largest quantification of whole blood linear and circular RNAs (circRNA) in early-stage idiopathic PD, using RNA sequencing data from two cohorts (PPMI = 259 PD, 161 Controls; ICICLE-PD = 48 PD, 48 Controls). We identified a replicable increase in *TMEM252* and *LMNB1* gene expression in PD. We identified novel differences in the expression of circRNAs from *ESYT2*, *BMS1P1* and *CCDC9*, and replicated trends of previously reported circRNAs. Overall, using circRNA as a diagnostic biomarker in PD did not show any clear improvement over linear RNA, minimising its potential clinical utility. More interestingly, we observed a general reduction in circRNA expression in both PD cohorts, accompanied by an increase in *RNASEL* expression. This imbalance implicates the activation of an innate antiviral immune response and suggests a previously unknown aspect of circRNA regulation in PD.

## Introduction

Parkinson’s disease (PD) is a neurodegenerative disease characterised by a progressive loss of dopaminergic neurons in the substantia nigra^1^. Diagnosis is predominantly based on the presentation of cardinal symptoms, including motor (such as bradykinesia and gait disturbances) and non-motor (sleep problems, cognitive impairment and dementia) symptoms^1^, which result in substantial morbidity^2^. Unfortunately, the clinical manifestation of PD typically occurs years after the onset of dopaminergic neuron loss, making early diagnosis challenging^3^. Measures to slow the rate of progression, therefore, remain a key goal in PD research^4^. The reliance on the observation of clinical symptoms hampers the detection in the earliest phases of neurodegeneration when disease-modifying intervention would be most beneficial^5^. Additionally, diagnostic accuracy is adversely affected by shared clinical presentations with other neurodegenerative conditions, resulting in an estimated misdiagnosis rate of ~25% in the early stages of disease^3^. The identification of biomarkers that can facilitate accurate early diagnosis and lead to the discovery of new therapeutic targets are therefore crucial for the future clinical management of PD, particularly in the early stages of disease^6^.

Despite significant effort, few PD biomarkers have been translated into clinical practice^6^, in part limited by a lack of replication^7^. Efforts to identify biomarkers of PD onset have been hampered by heterogeneity in clinical presentation and the rate of progression of patients^8^. Familial PD cases, both dominant and recessive, account for ~10–15% of cases^9^, whilst the majority of remaining cases are considered idiopathic. The current largest GWAS meta-analysis identified 90 variants explaining ~16–36% of PD heritability^10^, yet polygenic risk scoring is not currently at a stage to be used clinically to predict an individual’s risk^10^. Neuroimaging show promising results, with accuracies typically >80%^11^. However, these techniques are expensive and usually most accurate in advanced disease^12^ or when applied in combination^13^.

Biofluid-based biomarker candidates, including neurochemicals such as orexin^14^ and metabolites including lactate and acylcarnitines^15,16^ have been proposed. Recent work assessing α-synuclein seeding in CSF is promising in specific PD subgroups^17^, although the invasive nature of a lumbar puncture limits its utility. The accessibility of whole blood, as well as a high degree of overlap with neuronal expression (>80% shared gene expression)^18,19^, has prompted research into assessing blood-based RNAs as biomarkers of PD. Early array-based and, more recently, RNA sequencing-based experiments have identified several messenger RNA (mRNA)^20–28^, long non-coding RNAs (lncRNA)^29^ and microRNAs (miRNAs)^30–33^ as potential biomarkers of PD. However, there is little concordance between studies^24,26,34,35^, likely a result of differences in methodologies, sample cohorts and study design^24,26,36^.

**Fig. 1:**
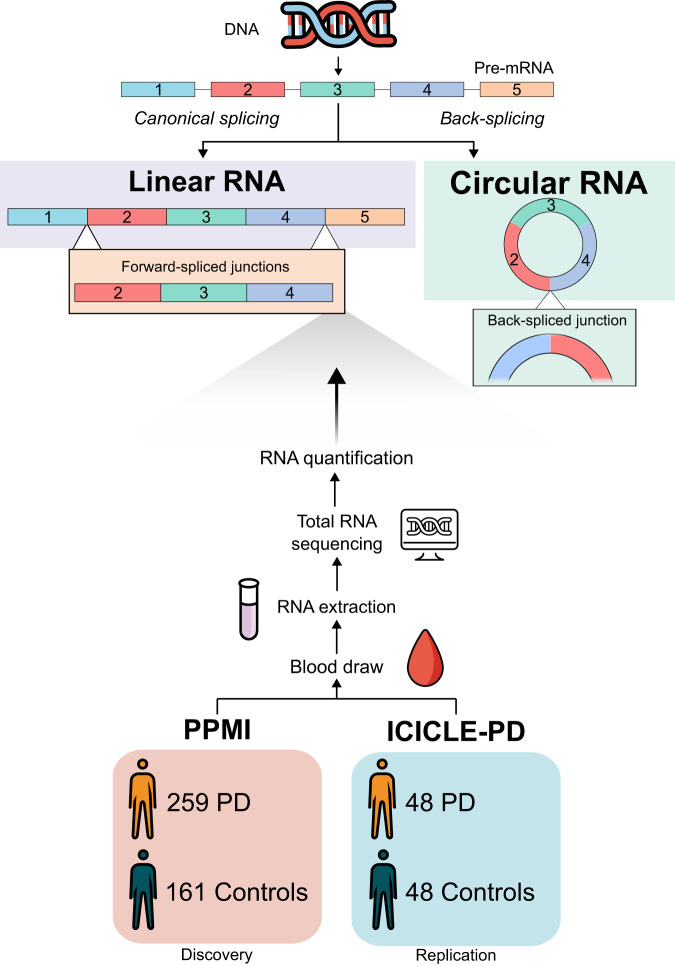
Circular RNA formation and study overview. The canonical splicing of pre-mRNA produces a linear RNA molecule containing forward-spliced junctions (FSJ). Circular RNA molecules are formed by a back-splicing reaction and can be identified through the presence of a back-spliced junction (BSJ). Individuals from our discovery (PPMI, PD = 259, Controls = 161) and replication (ICICLE-PD, PD = 48, Controls = 48) cohorts underwent whole blood total RNA sequencing to detect and quantify linear and circular RNAs.

Circular RNA (circRNA) is an RNA species characterised by the formation of a back-splice junction (BSJ, Fig. 1)^37^. CircRNA expression can vary across cell, tissue and development stages^38–40^. Interestingly, circRNA expression can be uncoupled from that of its parental host gene^40–42^ and several modulators of global circRNA levels have been reported^43–53^. CircRNAs are abundant in the brain^54^, enriched in genes related to neuronal function^55^, and readily identifiable in blood^40,56^. Their reported resistance to RNA exonuclease activity and increased stability compared to their linear counterparts^57,58^, have prompted investigations of their potential use as biomarkers in a range of human diseases, including PD^59,60^. Differential expression of circRNAs in PD has been reported^59–65^. However, these studies typically use small cohorts, identify specific circRNAs that are not replicated and show limited classification ability when compared to existing clinical predictors. Thus, a large-scale, unbiased assessment of circRNAs in PD is warranted.

Here, we investigate the potential utility of circRNA as biomarkers for early-stage idiopathic PD in two large unrelated cohorts, The Michael J Fox Foundation Parkinson’s Progression Markers Initiative^66^ (PPMI) and The Incidence of Cognitive Impairment in Cohorts with Longitudinal Evaluation-PD (ICICLE-PD)^67^, adopting a discovery-replication strategy. Our analysis indicates that, although circular RNAs and some genes are differently expressed, neither has sufficient discriminatory power to displace existing PD biomarkers. Interestingly, we observed a reproducible reduction in general circRNA expression levels in PD patients compared to controls, which was not recapitulated in canonical splice junction expression, suggestive of a link between circRNA expression and PD.

## Results

We utilised high-depth total RNA sequencing of whole blood in two unrelated PD cohorts to investigate circular RNA expression (Fig. 1). CircRNA expression does not always reflect host gene expression^40–42^, thus prior to analysing circRNAs, we compared gene expression (i.e., linear mRNA) between PD patients and controls in both PPMI and ICICLE-PD. We limited all our analysis to early-stage idiopathic Parkinson’s disease (PD) patients (i.e., diagnosed <13 months and no known predisposing genetic variation) to age and sex-matched controls, using PPMI as a discovery cohort and ICICLE-PD as a replication cohort (Fig. 1 and Supplementary Table 1).

### Differential gene expression in early-stage idiopathic PD

**Fig. 2:**
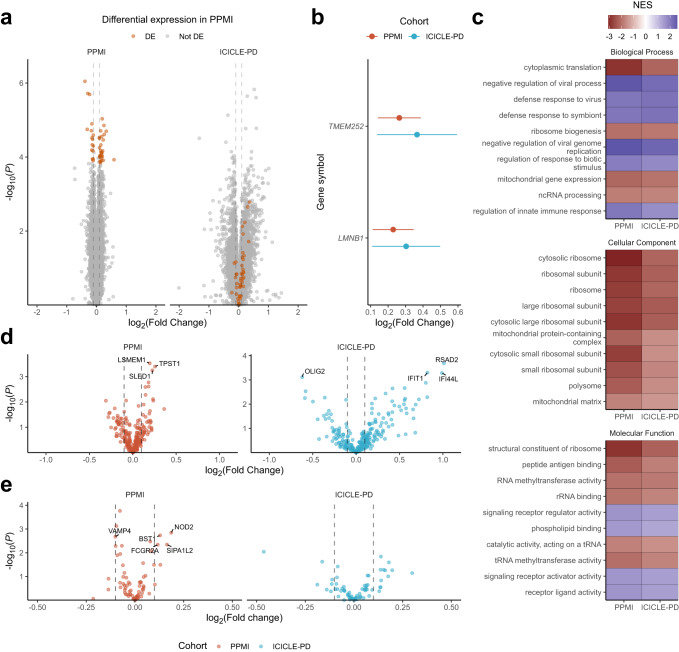
Gene differential expression in early-stage idiopathic PD. Figures showing the comparative expression of genes between PD cases and controls in PPMI and ICICLE-PD. **a** Volcano plot of differential expression in PPMI and ICICLE-PD, highlighted (orange) are the 44 genes significantly differentially expressed in PPMI (FDR < 0.05 and log_2_ fold change >0.1/ < −0.1) and the corresponding results in ICICLE-PD (Supplementary Data 1). **b** Graph showing the comparative log_2_ fold changes and 95% CI of genes (*TMEM252* and *LMNB1*) that were significant in both PPMI and ICICLE-PD. **c** Results from the gene set enrichment of Gene Ontology terms. Terms that were significantly enriched (FDR < 0.05) were replicated in ICICLE-PD. Displayed are the first 10 terms from each ontology as ranked by *P*-value in PPMI. NES = Normalised Enrichment Score (Supplementary Data 2). **d** Volcano plots showing the differential expression of genes reported as differentially expressed in PD from previous blood RNAseq studies^70–73^ (Supplementary Data 4). Genes significantly differentially expressed (FDR < 0.05 and log_2_ fold change >0.1/ < −0.1) in each cohort are labelled. **e** Volcano plots showing the differential expression of risk genes highlighted in PD GWAS^10^ (Supplementary Data 5). Genes significantly differentially expressed (FDR < 0.05 and log_2_ fold change >0.1/ < −0.1) in each cohort are labelled. Grey dashed lines on the volcano plots indicate the log_2_ fold changes 0.1 and −0.1.

Differential gene expression analysis was carried out on 16,191 (PPMI) and 15,852 (ICICLE-PD) genes respectively (overlap of 95.53%). In PPMI, we identified 44 significantly differentially expressed genes (log_2_FC < −0.1/ > 0.1, FDR < 0.05), with 28 upregulated and 16 downregulated in PD (Fig. 2a and Supplementary Data 1). Of these 44, *TMEM252* and *LMNB1* were similarly upregulated in ICICLE-PD (log_2_FC > 0.1, FDR < 0.05) (Fig. 2a, b and Supplementary Data 1).

To gain an overview of global alterations in gene expression patterns we performed gene set enrichment analysis (GSEA)^68^. After classifying into GO terms, we identified 97 biological processes, 11 molecular functions and 29 cellular components that were significantly enriched in PPMI and subsequently replicated in ICICLE-PD (FDR < 0.05 with the same direction of enrichment) (Supplementary Data 2). Ontologies related to ribosomal function and translation were more likely to be decreased in PD. In contrast, ontologies related to the innate immune response, including antiviral and interferon signalling processes, tended to be increased in PD (the top 10 ranked by PPMI *P*-value in each ontology are shown in Fig. 2c with all results present in Supplementary Data 2). GSEA of KEGG pathways also showed the significant enrichment of the pathways *Ribosome* (PPMI FDR = 7.58 × 10^−24^; ICICLE-PD FDR = 5.57 × 10^−10^) and *NOD-like receptor signalling* (PPMI FDR = 0.0016, ICICLE-PD FDR = 2.27 × 10^−6^), part of the innate immune response^69^ (Supplementary Data 3).

We were able to independently replicate (FDR < 0.05) genes previously reported as differentially expressed in the blood^70–73^ using PPMI (*LSMEM1*, *TPST1* and *SLED1*) and ICICLE-PD (*IFIT1*, *RSAD2*, *IFI44L* and *OLIG2*) datasets (Fig. 2d and Supplementary Data 4). Overlapping the expression of genes potentially underpinning PD GWAS risk loci^10^, identified five significantly differentially expressed genes (*BST1*, *FCGR2A*, *SIPA1L2*, *NOD2* and *VAMP4*, log_2_FC > 0.1/ < −0.1, FDR < 0.05) in PPMI, although none passed multiple testing correction in ICICLE-PD (Fig. 2e and Supplementary Data 5). Overlapping with highly penetrant pathogenic parkinsonism genes, we identified *PTRHD1* as significantly decreased in PD (FDR = 0.015), with a similar trend reported in ICICLE-PD (FDR = 0.088) (Supplementary Data 6).

### CircRNAs are differentially expressed in early-stage idiopathic PD

**Fig. 3:**
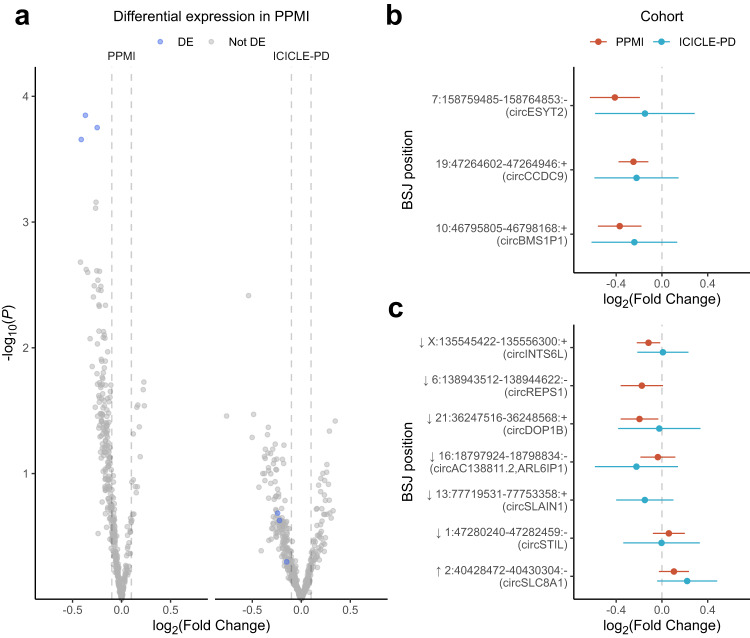
Circular RNA differential expression in early-stage idiopathic PD. Figures showing the comparative expression of circular RNAs (shown as individual back-spliced junctions, BSJs) between PD cases and controls in PPMI and ICICLE-PD. **a** Volcano plot of differential expression in PPMI and ICICLE-PD, highlighted (blue) are the three BSJs significantly differentially expressed in PPMI (FDR < 0.05 and log2 fold change >0.1/ < −0.1) and the corresponding results in ICICLE-PD (Supplementary Data 8). Grey dashed lines indicate the log_2_ fold changes of 0.1 and −0.1. **b** Graph showing the comparative log_2_ fold changes of the three BSJs that were significantly differentially expressed in PPMI. None reached significance in ICICLE-PD (*P*-values > 0.05) (Supplementary Data 8). **c** Graph showing the comparative log_2_ fold changes of BSJs previously reported as being differentially expressed in PD^61,62^ (Supplementary Data 9). The arrows indicate the previously reported direction of change in PD relative to controls. Error bars in **b** and **c** show the 95% CI of the fold change. BSJ positions are reported as chromosome:start-end:strand (GRCh38).

CircRNAs were detected based on the presence of a back-spliced junction (BSJ, Fig. 1), combining multiple detection algorithms to define high-confidence BSJs (see *Circular RNA detection and quantification*). Filtering of lower expressed circRNAs left a set of abundant circRNAs (PPMI = 403, ICICLE-PD = 457, 62.57% overlap). We observed no overrepresentation of GO terms after grouping circRNAs based on their host gene (Supplementary Data 7). We identified three BSJs significantly reduced in PD patients versus controls in PPMI (log_2_FC < −0.1/ > 0.1, FDR < 0.05) (Fig. 3a, b and Supplementary Data 8). These BSJs, derived from the genes *ESYT2*, *BMS1P1*, and *CCDC9* were similarly decreased in ICICLE-PD but did not reach statistical significance (FDR > 0.05) (Fig. 3a, b and Supplementary Data 8). Seven circRNAs previously reported as differentially expressed in PD^61,62^ were sufficiently expressed in the PPMI or ICICLE-PD cohorts (Supplementary Data 9). Two BSJs, derived from *DOP1B* and *INTS6L*, showed altered expression in PPMI (*P*-value < 0.05) (Supplementary Data 9). While no BSJ reached statistical significance after multiple testing correction (FDR > 0.05), we were able to replicate the direction of change of four BSJs in PPMI and three in ICICLE-PD (Fig. 3c and Supplementary Data 9).

### Early-stage idiopathic PD is characterised by a reduction in circRNA abundance

**Fig. 4:**
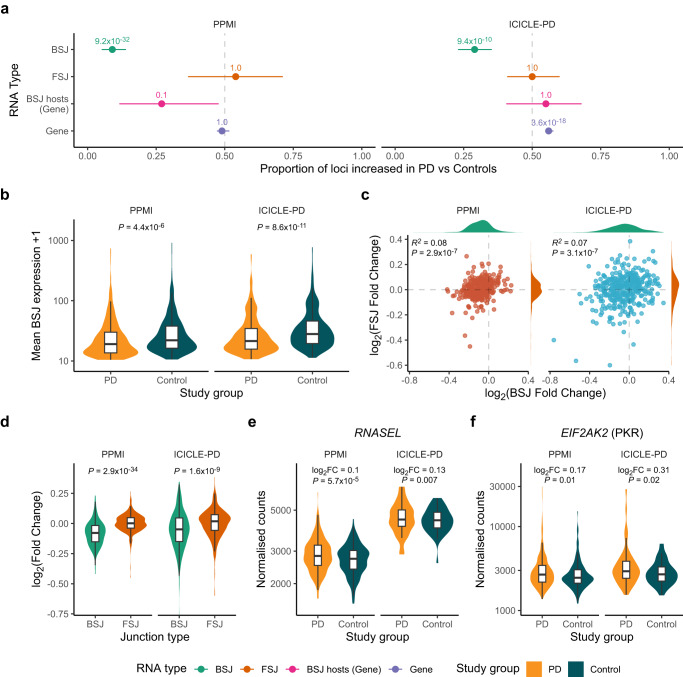
Idiopathic PD patients exhibit a circular RNA-specific reduction in expression. **a** Proportion of loci increased in PD relative to controls based on differential expression testing of back-spliced junctions (BSJs), forward-spliced junctions (FSJ), genes which harbour a BSJ (BSJ hosts) and all genes (Gene). *P*-values obtained from a two-sided exact binomial test corrected for multiple testing (Bonferroni correction, four tests). Error bars show the 95% CI of the imbalance estimate. **b** Comparing the expression of all BSJs included in differential expression testing between PD and controls in PPMI and ICICLE-PD. *P*-values are derived from a Wilcoxon rank-sum test comparing BSJ expression between PD and controls. **c** Correlation between BSJ and FSJ expression fold changes in both PPMI and ICICLE-PD. The correlation coefficients are reported as Spearman’s rho. Density plots show the distribution of the fold changes for each respective junction type. **d** Comparison of fold changes between BSJs and FSJs in PPMI and ICICLE-PD. *P*-values derived from a Wilcoxon rank-sum test comparing fold changes between each junction type. **e**, **f** Plots showing the normalised expression of the genes *RNASEL* and *EIF2AK2* (which encodes PKR) in PD and controls from PPMI and ICICLE-PD. Log_2_ fold changes and *P*-values were obtained from the results of testing the differential expression of genes.

Whilst we observed differential expression of specific BSJs, perhaps the more striking observation was an imbalance of BSJ fold changes, i.e., where the expression of the majority of BSJs tended to be reduced in PD compared to controls (PPMI imbalance = 0.09, Bonferroni *P*-value = 9.2 × 10^−32^, ICICLE-PD imbalance = 0.29, Bonferroni *P*-value = 9.4 × 10^−10^, Exact binomial test) (Fig. 4a, Supplementary Data 10). To explore whether reduced expression was specific to BSJs, we also examined the expression of other RNA types (Supplementary Fig. 7, Supplementary Data 10). Forward-spliced junction (FSJ) counts provide the best approximation of the linear RNA as gene expression estimates will inevitably include circRNA-derived reads that do not span the BSJ and thus cannot be distinguished. Crucially, a reduction was not evident when examining the fold changes of FSJs (PPMI imbalance = 0.54, Bonferroni *P*-value = 1.0, ICICLE-PD imbalance = 0.50, Bonferroni *P*-value = 1.0, Exact binomial test) (Fig. 4a, Supplementary Data 10). A comparison of fold changes based only on genes that host circRNAs identified no reduction (PPMI imbalance = 0.27, Bonferroni *P*-value = 0.1, ICICLE-PD imbalance = 0.54, Bonferroni *P*-value = 1.0, Exact binomial test). Global gene expression also showed no reduction in PPMI (imbalance = 0.49, Bonferroni *P*-value = 1.0), yet was significantly increased in ICICLE-PD (imbalance = 0.56, Bonferroni *P*-value = 3.6 × 10^−18^) (Fig. 4a, Supplementary Data 10).

As fold change imbalance estimates are dependent on the number of RNAs passing the fold change threshold, we also directly compared expression levels of all RNAs included in differential expression analyses. This also provided evidence of a significant reduction in circRNA expression in PD (PPMI *P*-value = 4.4 × 10^−6^, ICICLE-PD *P*-value = 8.6 × 10^−11^, Wilcoxon rank-sum test) (Fig. 4b), yet no reduction in the expression of flanking FSJs, expression of circRNA host genes or total gene expression (Supplementary Fig. 8).

To highlight differences in circRNA regulation, we compared fold changes between BSJ expression and their flanking FSJs. For a subset of junctions, this was not possible as no corresponding FSJs were detected (PPMI = 95/403 or 23.6%, ICICLE-PD = 92/457 or 20.1%). We observed a weak correlation in fold change differences between BSJs and corresponding FSJs (PPMI R^2^ = 0.08, ICICLE-PD R^2^ = 0.07, Fig. 4c). BSJ fold changes were consistently lower, albeit generally modest, across both cohorts (PPMI *P*-value = 2.9 × 10^−34^, ICICLE-PD *P*-value = 1.6 × 10^−9^, Wilcoxon rank-sum test) (Fig. 4d). Together, these results suggest that the observed imbalance in circRNA expression in early-stage idiopathic PD is not solely driven by a reduction in parental host gene expression.

### *RNASEL* and *ADAR* expression is increased in early-stage idiopathic PD

CircRNA biogenesis and degradation are linked to several *cis-* and *trans*-acting factors^37^. We explored whether differential expression of previously reported circRNA regulators^43–53^ may be contributing to the reduced circRNA abundances in PD (regulators and evidence are given in Supplementary Data 11). In PPMI, *RNASEL* and *ADAR* were significantly increased in PD, with *RNASEL* also increased in ICICLE-PD and significant after multiple testing correction (Fig. 4e, Supplementary Data 11). *RNASEL* encodes Ribonuclease L, which is known to be involved in the degradation of circRNA, subsequently allowing the activation of PKR as part of the integrated stress response^50^. We also observed significantly increased expression of *EIF2AK2*, which encodes PKR, in both cohorts (PPMI *P*-value = 0.010; ICICLE-PD *P*-value = 0.019) (Fig. 4f, Supplementary Data 1). Increased PKR activity has been reported in lymphocytes of PD patients^74^. This, combined with the downregulation of protein synthesis genes and the upregulation of genes involved in the innate immune response (Fig. 2c, Supplementary Data 2), suggests that the antiviral integrated stress response may be active in early-stage idiopathic PD.

### Evaluating RNA expression as a predictor of early-stage idiopathic PD

**Fig. 5:**
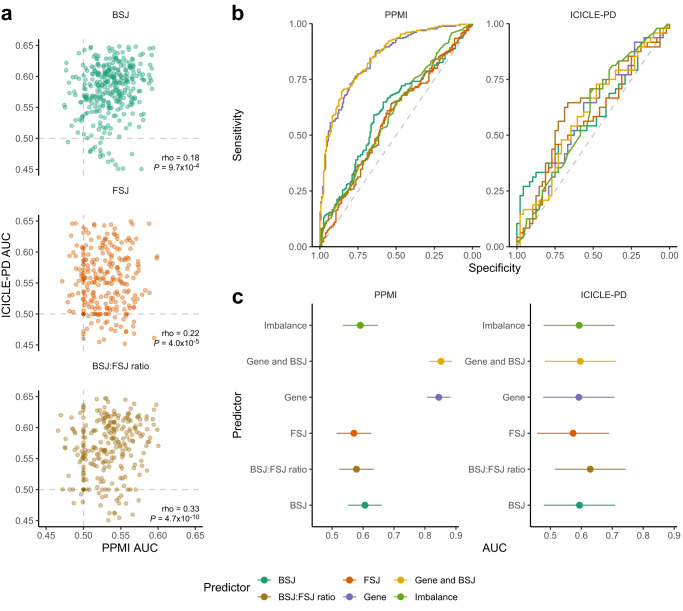
Using RNA expression to classify early-stage idiopathic PD status. **a** Comparison of AUCs calculated in PPMI and ICICLE-PD cohorts using the expression of individual junctions (BSJ and FSJ) and BSJ:FSJ ratios to classify PD status. ROC curves were constructed using the expression of back-spliced junctions (BSJ, VST normalised), forward-spliced junctions (FSJs, VST normalised) and the circular to linear ratio (BSJ:FSJ ratio, calculated by CIRIquant) for each junction position. Spearman’s rho demonstrates the correlation between the AUC in each cohort. Grey dashed lines indicate an AUC of 0.5. **b**, **c** Ability of each predictor to classify PD status. ROC curves (**b**) showing the sensitivity and specificity of each predictor (see methods) at various thresholds in PPMI and ICICLE-PD cohorts. The area under the ROC curve for each predictor is shown in **c**. Performance in the PPMI was evaluated on held-out data in the outer nested folds. Performance in ICICLE-PD was based on models fit on the PPMI data. Error bars give the 95% confidence intervals of the AUC.

To evaluate the use of RNA expression as a predictor of early-stage idiopathic PD, we first examined the potential of BSJ and FSJ expression, as well as BSJ:FSJ expression ratios, to separate PD from controls. Area under the receiver operating characteristics curves (AUC) were generated using the expression of each abundant junction type as a predictor. The median AUC across all junction measures was higher in ICICLE-PD (0.57) compared to PPMI (0.53). The strongest correlation between PPMI and ICICLE-PD AUCs was for BSJ:FSJ ratios (Spearman’s ρ = 0.33), followed by FSJ (Spearman’s ρ = 0.22) and BSJ counts (Spearman’s ρ = 0.18) (Fig. 5a).

With no clear individual junction biomarker candidates, we included multiple features in multivariable regularised logistic regression models (see *Classification of Parkinson’s status using RNA abundances)*. Using cross-validation, we trained and evaluated the classification performance of models using the PPMI cohort. We then used the ICICLE-PD as an independent test cohort to assess the performance of classifiers trained using the PPMI cohort. The best performance in the PPMI cohort was achieved by combining gene and BSJ counts (AUC = 0.85 [0.81, 0.89], Fig. 5b) and appeared primarily driven by gene expression (AUC = 0.84 [0.81, 0.88], Fig. 5b) rather than BSJ counts (AUC = 0.61 [0.55, 0.66], Fig. 5b). In ICICLE-PD, combining gene and BSJ counts produced an AUC of 0.60 [0.48, 0.71] (Fig. 5b). Similarly, the performance of the gene expression strategy also decreased in the ICICLE-PD cohort (AUC = 0.59 [0.48, 0.71], Fig. 5b). All other classifiers had an AUC < 0.6 in the PPMI cohort (Fig. 5b, Supplementary Table 2). Using BSJ:FSJ ratio as a classifier achieved the best generalisability in the ICICLE-PD cohort (AUC = 0.63 [0.52, 0.74], Fig. 5b). All other unmentioned models achieved an AUC < 0.6 in the ICICLE-PD cohort (Fig. 5b, Supplementary Table 2).

## Discussion

We identified differences in gene and circRNA expression between early-stage idiopathic PD patients and controls, with elevated expression of *TMEM252* and *LMNB1* observed in both PPMI and ICICLE-PD PD cases compared to controls. We independently validated expression changes in genes shown to modulate PD risk^10^ and in genes that had been previously reported to be differentially expressed in PD^70–73^. Additionally, we discovered three novel circRNAs that were decreased in PD, with similar trends observed in our replication cohort. After evaluating the performance of multivariable classification models, our results suggest that circRNAs are unlikely to be useful biomarkers for early-stage idiopathic PD, particularly when compared to classification using gene expression. Perhaps more interestingly, we observed a tendency towards generalised decreased circRNA expression in PD in both cohorts, which correlated alongside decreased expression of genes involved in protein translation and increased expression of genes involved in the immune response. These findings suggest that reduced circRNA levels in the blood may be a proxy measure of the underlying pathology and add to the growing body of evidence linking RNA metabolism and immune response to PD^75,76^.

**Fig. 6:**
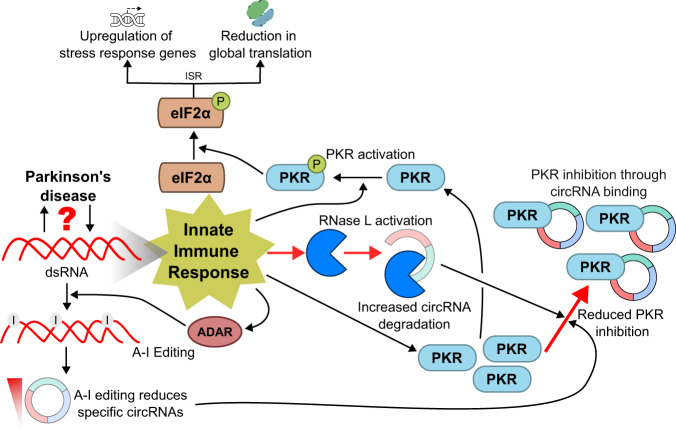
A mechanism of circRNA reduction in Parkinson’s disease. Innate immune responses can be induced by double-stranded RNA (dsRNA). A-I RNA editing by ADAR suppresses immune detection of endogenous dsRNA^149^ and influences circRNA synthesis^53,54,77,78^. Under normal conditions, circRNAs bound to PKR (encoded by *EIF2AK2*) act as inhibitors, undergoing degradation when activation is required^50^. Activation of the endonuclease RNase L as part of an antiviral immune response leads to degradation of circRNAs^50^. Decreased circRNAs in idiopathic PD could result in increased PKR, available for activation. Once activated, PKR phosphorylates eIF2α, initiating the integrated stress response (ISR)^81^.

The global downregulation of circRNA expression we observed in blood is reminiscent of the reduction in microRNA expression seen in PPMI participants^33^. CircRNA expression is influenced by trans-acting proteins, including Ribonuclease L (RNase L, encoded by *RNASEL*)^50^ and adenosine deaminase RNA specific (ADAR, encoded by *ADAR*), an enzyme responsible for A-I RNA editing that can modulate the base-pairing of reverse complementary matches in the flanking introns of circRNAs, influencing their biogenesis^53,54,77,78^. *ADAR* was significantly increased in PPMI PD cases and showed a similar trend in ICICLE-PD (Supplementary Data 11). Although *ADAR* expression levels do not necessarily correlate with editing activity^79^, altered RNA editing has been observed in PD patients’ blood and brain regions^61,80^. RNase L degrades circRNAs as a response to viral infection^50^. The degradation of circRNAs is required for the activation of Protein Kinase R (PKR, encoded by *EIF2AK2*), a regulator of the integrated stress response (ISR)^50,81^. This is consistent with our results, where the reduction in circRNAs in PD we observed coincides with increased expression of *RNASEL* and *EIF2AK2* (Fig. 4e, f, Supplementary Data 1), as well as the upregulation of genes related to antiviral activity and the innate immune response (Fig. 2c, Supplementary Data 2, 3). Although the role of RNase L in the antiviral immune response is well documented^82^, and despite the links between viral infection and PD^83^, to our knowledge, there have been no reports showing *RNASEL* expression changes in PD. Conversely, PKR activation has been observed in PD^74,84^. Endogenous dsRNA can lead to PKR activation^85,86^ and has been recently implicated in neurodegenerative diseases other than PD^87–89^. In mice, exogenous dsRNA triggered α-synuclein aggregation and dopaminergic neuron loss, suggesting the existence of aberrant dsRNA may have a potential role in the pathogenesis of PD^90^. Activated PKR leads to eIF2α phosphorylation, and results in global protein synthesis attenuation^81^. Protein synthesis is reduced in PD patient cell lines^91,92^, consistent with the reduced expression of genes related to protein synthesis and ribosomal function we observed in PD (Fig. 2c, Supplementary Data 2, 3). Based on our findings, we propose that reduced circRNA levels in early-stage idiopathic PD is related to activation of an antiviral immune response, reflected through increased expression of *RNASEL*, *ADAR* and *EIF2AK2* (which encodes PKR) (Fig. 6). Reduced circRNA expression would allow activation of PKR, consistent with the increased activity of PKR in lymphocytes of PD patients^74^, triggering the cellular ISR. Overall, these results highlight a potential role of RNase L and PKR in the pathogenesis and/or systemic response to PD (Fig. 6).

Using gene expression as a classifier of early-stage PD in the PPMI cohort was in line with previously published estimates (AUC = 0.84 compared to 0.80 in Makarious et al. ^93^, Fig. 5c). In our independent replication cohort (ICICLE-PD), the AUC of the gene expression classifier was lower (ICICLE-PD AUC = 0.59, Fig. 5c). Despite promising results using circRNA as a biomarker in other diseases^59^, circRNAs performed substantially worse than gene expression when classifying PD in PPMI (AUC = 0.61 vs 0.84, Fig. 5c), although similar performance was seen in ICICLE-PD (AUC = 0.59 vs 0.59, Fig. 5c). Similar to previous work assessing circRNAs as a lung cancer biomarker^94^, combining circRNA expression with gene expression showed a small improvement in predictive ability over gene expression alone in both PPMI (AUC = 0.85 vs 0.84, Fig. 5c) and ICICLE-PD (AUC = 0.60 vs 0.59, Fig. 5c). Put into context, circRNAs perform worse than their linear counterparts when classifying PD as a group, leading us to the conclusion that circRNA expression in isolation has limited utility as a clinical PD biomarker. Although combining circRNAs and gene expression data does improve classification power, the performance of circRNAs either in isolation or combined with gene expression falls short of recent estimates using alpha-synuclein seeding as a biomarker^17^.

When comparing differential gene expression, we identified two genes (*TMEM252* and *LMNB1*) that were upregulated in PD cases in both cohorts, consistent with previous work^27^. *TMEM252* encodes a transmembrane protein of unknown function that has been linked to cancer^95^, but has not previously been implicated in PD. *LMNB1* encodes Lamin B1, a component of the nuclear lamina. Increased *LMNB1* expression has been reported in the dopaminergic neurons of PD patients^96^ and a variant within *LMNB1* has been associated with cognitive outcomes in PD^97^.

We were also able to independently replicate the differential expression of genes that have been previously reported in blood RNA-seq studies as differentially expressed in PD patients^70–73^ (*LSMEM1*, *TPST1* and *SLED1*, Fig. 2d). As previous studies were not limited to early-stage PD (diagnosed <13 months), these genes may reflect those with altered expression throughout PD development. Of the three genes replicated using PPMI, *SLED1* and *LSMEM1* encode proteins of unknown function. *TPST1* encodes tyrosylprotein sulfotransferase 1, an enzyme required for post-translational modification of proteins that also plays important roles in the inflammatory process, leukocyte movement and cytosis, viral cell entrance, and other cell-cell and protein-protein interactions^98^. Interestingly, 3 out of the 4 previously reported genes replicated in ICICLE-PD (*IFIT1*, *RSAD2*, *IFI44l*, Fig. 2d) are induced by type-1 interferons^99^. Their increase in expression is consistent with an increase in type-1 interferon signalling in PD^100^. In addition, our data provide a potential mechanistic link between PD-associated genetic variation, identifying differential expression among genes associated with PD risk. These included *PTRHD1* (Supplementary Data 6), which encodes a peptidyl-tRNA hydrolase that has been linked to recessive parkinsonism^101,102^, and genes identified by association studies (*BST1*, *FCGR2A*, *SIPA1L2*, *NOD2* and *VAMP4*, Fig. 2e)^10^.

Our analysis of circRNAs identified three that were over-expressed in PPMI PD cases compared to controls (within the genes *BMS1P1*, *CCDC9* and *ESYT2*) with similar trends observed in ICICLE-PD (Fig. 2a, b). These circRNAs, or their host genes, have not previously been associated with PD. The protein product of *CCDC9* is believed to be a member of the exon junction complex involved in RNA splicing^103^. CircRNAs from *CCDC9* have been implicated in cancer, acting as a miRNA sponge to suppress tumorigenesis^104^, and in stroke, suppressing NOTCH signalling in mouse models of ischaemia^105^. Interestingly, alterations in NOTCH signalling has been linked to PD through the function of LRRK2^106^. *ESYT2* encodes extended synaptotagmin 2, a member of the E-Syt family, which are endoplasmic reticulum (ER) localised proteins involved in tethering the ER to the cellular plasma membrane^107^. There is suggestive evidence that circRNAs derived from *ESYT2* may be upregulated upon viral infection^108^, in contrast to the decreased expression we observed in PD patients (Fig. 2a, b). *BMS1P1* encodes a pseudogene of ribosome biogenesis factor pseudogene 1 (BMS1), and there are no known disease associations for circRNAs produced by this gene.

We were also able to independently replicate trends in several previously reported differentially expressed circRNAs observed in PD blood and brain tissue^61,62^ although none reached statistical significance (Fig. 3c, Supplementary Data 9). This is likely due to methodological differences (e.g., circRNA detection and normalisation), tissue-specific expression (whole blood compared to the brain) or differences in blood cell composition (whole blood compared to peripheral blood mononuclear cells). Unfortunately, 23 of the 30 previously reported circRNAs (20 from the substantia nigra and three detected in peripheral blood mononuclear cells) were undetectable in either PPMI or ICICLE-PD. We identified altered expression of circRNAs (derived from *DOP1B* and *INTS6L*), whose host genes have not been functionally linked to PD (Supplementary Data 9). However, copy number variation in *DOP1B* has previously been linked to Alzheimer’s disease^109^, while deletion of *INTS6L* leads to a cardiomyopathic phenotype^110^. Nonetheless, our data extend the growing body of evidence linking circRNA dysregulation to PD^60^.

As a key vehicle for immune cells, the differences we observe in blood RNA expression in PD patients add to the proposed role of inflammation and immune dysfunction in the development and response to PD^76^. It is possible that changes in blood can be detected early on in disease duration, in line with the early presentation of non-motor symptoms and peripheral aggregations of α-Synuclein^111^. Pre-diagnosis changes in lymphocyte levels and function have been reported^112,113^. In addition, inflammation markers within blood have been associated extensively with both PD risk and symptom progression^114^.

In this work, we have drawn together and performed the largest whole-blood circular transcriptome analysis of PD, focusing on the potential utility of circRNAs as biomarkers. However, several limitations must be considered. Biomarker detection using whole-blood has numerous advantages, including accessibility and ease of processing, but we could not account for variation in the proportions of different blood cell types across samples^115,116^. In addition, despite efforts to increase homogeneity (see *Cohorts*), both cohorts differ in some respects. Firstly, PPMI participants were not receiving treatment at sample collection, whereas most ICICLE-PD participants were. Dopaminergic treatment may influence biological measures, such as circulating cell-free mitochondrial DNA levels^117^. It must also be noted that some PPMI samples were recruited during a treatment interval (up to ~60 days before enrolment) and a large number of individuals were taking concomitant medications for a range of non-PD and PD-related symptoms^66^. This may have influenced our results, however, the concordances we see between RNA expression as well as the reproducible reduction in circRNA expression in PD patients between cohorts suggests this is minimal. Secondly, it is inevitable that the difference between cohort sizes affected our ability to detect rarer RNAs, particularly circRNAs. We attempted to mitigate against this by using the largest cohort as discovery (PPMI) opting to replicate in the smaller cohort (ICICLE-PD). In addition, we limited our analysis to abundant genes and circular RNAs, with the majority (95.53% of genes and 62.57% of circRNAs) detectable in both cohorts. Finally, some circRNA quantification studies use circRNA enrichment steps such as RNase R treatment^118^ to enhance the detection of lesser expressed circRNAs. However, not depleting linear RNAs allowed us to quantify both linear and circular RNAs simultaneously.

In conclusion, we observed specific and consistent alterations in the linear and circular blood transcriptome in early-stage idiopathic PD patients. Changes in circRNA levels were not sufficient to facilitate reliable PD classification, particularly when compared to existing PD biomarkers. We did, however, identify a reproducible reduction in circRNA expression in PD. This imbalance, along with gene expression patterns, implicates the activation of an innate antiviral immune response, providing an opportunity for future investigations into this previously unknown aspect of circRNA regulation in PD.

## Methods

### Cohorts

We utilised samples with corresponding demographic (e.g., age at sample collection, sex) and clinical data (e.g., dopaminergic treatment status, disease duration) from two large cohorts of patients with Parkinson’s disease (PD) and controls of similar ages and sex. The discovery cohort was obtained from The Michael J Fox Foundation Parkinson’s Progression Markers Initiative^66^ (PPMI, https://www.ppmi-info.org/), while The Incidence of Cognitive Impairment in Cohorts with Longitudinal Evaluation-PD^67^ (ICICLE-PD, https://www.bam-ncl.co.uk/iciclepd) was used for replication. To study transcriptomic changes in the early stages of PD and to ensure parity between discovery and replication cohorts, only PD patients recently diagnosed with PD (<13 months) were included. PD patients harbouring causative variants in select genes (e.g., *LRRK2*, *GBA*, *SNCA*, *PINK1*, *PRKN*) were excluded and are thus all PD cases are idiopathic. Both studies were conducted in accordance with the Declaration of Helsinki and Good Clinical Practice guidelines after approval of local ethics committees of the participating sites (PPMI, https://www.ppmi-info.org/ and ICICLE-PD, Newcastle and North Tyneside Research Ethics Committee)^66,67^. All subjects provided written informed consent.

### ICICLE-PD RNA isolation and sequencing

Between June 2009 and December 2011, newly diagnosed PD patients were recruited from the community and hospital outpatient clinics in Newcastle-upon-Tyne and Gateshead. Idiopathic PD was diagnosed by a movement disorder specialist and fulfilled Queen’s Square Brain Bank criteria^119^. Full exclusion criteria have been published elsewhere^67^. Briefly, participants were excluded if they had significant cognitive impairment at presentation (Mini Mental State Examination <24) or a pre-existing diagnosis of dementia, an atypical Parkinsonian syndrome, or insufficient English to complete assessments. We limited our analysis to 48 early-stage (diagnosed <13 months) idiopathic (i.e., without a known PD-related genetic diagnosis) PD patients and 48 control samples matched for age and sex. Total RNA was extracted from 5 ml of whole blood collected and stored in PAXgene tubes as per manufacturer’s instructions (Blood RNA Kit, Qiagen), quality assessed using an Agilent 2100 Bioanalyzer system and stored at −80 °C. Only samples with an RNA integrity number >8 were included. Library preparation, including ribosomal RNA and globin depletion (Globin-Zero Gold rRNA Removal Kit, Illumina), was carried out using the TruSeq Stranded Total RNA kit (Illumina). Sequencing was performed using an Illumina NovaSeq 6000 generating 150 bp paired-end reads. Median sequencing depth was estimated as 89.2 (IQR = 15.7) million paired-end reads (Supplementary Fig. 1).

### PPMI RNA isolation and sequencing

Subject recruitment and eligibility criteria for the PPMI study have been previously published^66^. To increase matching between ICICLE-PD, we limited our analysis to 287 early-stage (diagnosed <13 months) idiopathic (i.e., without a known PD-related genetic diagnosis) PD patients and 176 controls, additionally matching within PPMI for age and sex. Whole blood RNAseq data was downloaded as FASTQ files from the online PPMI repository (https://www.ppmi-info.org/). RNA collection, isolation and subsequent sequencing have been previously reported^27^ and is described in the PPMI Biologicals Manual. Briefly, whole-blood RNA was extracted from PAXgene tubes (Blood RNA Kit, Qiagen), rRNA and globin depleted (Globin-Zero Gold rRNA Removal Kit, Illumina). Library preparation used the NEB/Kapa (NEBKAP) kit (see ref. ^27^ for more information). Sequencing was performed using an Illumina NovaSeq 6000 generating 125–150 bp paired-end reads. Median sequencing depth was estimated as 107 (IQR = 31.2) million paired-end reads (Supplementary Figure 1).

### RNA alignment and quantification

FASTQ files were aligned to the human genome (Ensembl GRCh38^120^) using HISAT2 v2.1.0^121^. RNA transcripts were quantified using the Ensembl GRCh38 v101 cDNA reference and Salmon v1.3.0^122^ using the ‘selective alignment mode’ option and correcting for sequence-specific (*--seqBias*), GC-content (*--gcBias*) and positional biases (*--posBias*). Transcript counts were translated into gene-level counts using tximport v1.26.1^123^ and annotated using the Ensembl v101 GRCh38 reference.

### Circular RNA detection and quantification

Reads derived from circular RNA (circRNA) molecules were identified by the presence of a back-spliced junction (BSJ, Fig. 1) detected using three tools: CIRI2 v2.0.6^124^, PTESfinder v2.0^125^ (https://github.com/osagiei/pfv2) and CIRCexplorer v2.3.8^126^. First, FASTQ files were quality (Phred <15) and then adapter trimmed using Trim Galore v0.67 running cutadapt v4.2^127^. Reads were aligned to the human genome (Ensembl GRCh38) according to each tool’s specification: STAR v.2.7.10a^128^ for CIRCexplorer2 and PTESfinder v2, bowtie v2.3.4^129^ for PTESfinder v2, and BWA v0.7.17^130^ for CIRI2. All BSJs were annotated using the Ensembl v101 GRCh38 reference.

Similar to others^56^, we limited false positives by retaining BSJs with a read count >1 in at least two individuals. In addition, BSJs were only retained for downstream analysis if they were detected by at least two tools (Supplementary Figure 2). This reduced our initial set of BSJs (PPMI = 438,189 to 23,454, or 5.35%; ICICLE-PD = 222,133 to 15,345, or 6.91%). These BSJs, alongside the corresponding forward-spliced junctions (FSJs, Fig. 1), were quantified with CIRIquant v1.1.2^131^ using HISAT v2.2.0^121^ as the aligner.

### Sample level quality control

Sample level quality control was assessed in both cohorts to identify sample failure, RNA contamination and abnormal global transcriptome issues. Fastq Screen v0.14.1^132^ and FastQC v0.11.7 (https://www.bioinformatics.babraham.ac.uk/projects/fastqc/) were used to assess contamination and obtain general sequencing metrics. Alignment metrics were obtained using *CollectRnaSeqMetrics* from Picard v2.27.5 (http://broadinstitute.github.io/picard/) and *stats* from SAMtools v1.6^133^. Additionally, PPMI samples that had been previously flagged due to QC issues were removed^27^. Validation of each participant’s clinically recorded sex was based on the normalised and variance-stabilising transformed^134^ expression of the Y chromosomal genes *RPS4Y1*, *KDM5D*, *DDX3Y* and *USP9Y*. Principal component analysis was carried out and the first two principal components and was used to detect mismatches by visual inspection (Supplementary Fig. 3). This identified one incorrectly coded individual in ICICLE-PD, which was corrected for analysis. After QC, the final PPMI dataset comprised of 259 PD and 161 controls; the ICICLE-PD dataset comprised of 48 PD patients and 48 controls (summarised in Supplementary Table 1). We observed no significant differences between the age or sex profiles of cases and controls in either dataset (Supplementary Table 1).

### Identification of sources of variation in RNA expression data

Biological and technical factors are known to impact the quantification of gene expression^135,136^. Like previous large-scale transcriptomic studies, we quantified sources of expression variation at both the sample and RNA level^27,137,138^. At the sample level, we used univariate linear regression to identify technical sequencing metrics (obtained from Picard and SAMtools) that explained a high proportion of the variance (R^2^ > 0.5) associated with the first 10 principal components of gene and circRNA expression (Supplementary Figs. 4a, b and 5a, b). At the gene level, based on recommendations^139^, we excluded highly correlated factors (Spearman’s ρ > 0.9, Supplementary Figs. 4c and 5c), subsequently quantifying the contribution of this reduced set of covariates to gene and circRNA expression variation using variancePartition v1.28.3 (Supplementary Figure 6). This final set of cohort-specific covariates were then included in regression modelling (see *Differential expression*).

### Differential expression

Normalisation and differential expression analysis of genes (i.e., linear mRNA) and BSJs was carried out using DESeq2 v1.38.2^140^. Gene and BSJ raw counts were filtered for low counts (>10 counts in the smallest sample group). Gene counts were library normalised using the default median-of-ratios method in DESeq2 (Anders and Huber, 2010). Junction counts (both BSJs and FSJs) were normalised using the sample size factors generated during the normalisation of gene counts.

Differential expression between PD and controls was assessed using a Wald test in DESeq2, adjusted for specific technical and biological covariates relevant to each cohort and RNA type (see *Identification of sources of variation in gene expression data*). Across all cohorts and RNA types, we adjusted for sex, age of collection and sequencing batch. For gene differential expression, we adjusted for the percentage of usable bases (PPMI), in addition to the percentage of coding and intronic bases (ICICLE-PD). For circRNA differential expression, we adjusted for the percentage of intronic bases (PPMI and ICICLE-PD), in addition to the median Coefficient of Variance of transcript coverage (ICICLE-PD). *P*-values were adjusted for multiple significance by the Benjamini-Hochberg procedure at a false discovery rate (FDR) of 5%^141^. Similar to previous work^27^, significantly differentially expressed genes and BSJs were defined as those that had an FDR < 0.05 and a log_2_ fold change (log_2_FC) <−0.1/ > 0.1.

### Ontology and pathway analysis

Enrichment of gene sets was performed using clusterProfiler v4.6.2^142^. Gene set enrichment analysis (GSEA)^68^ was based on gene lists ranked by fold change. For each cohort, all genes included in differential expression testing were used as the background set. Gene sets were obtained as Gene Ontologies^143^ (including biological processes, molecular functions, and cellular compartments) and KEGG pathways^144^ using the *gseGO* and *gseKEGG* functions, respectively.

GSEA is not suitable for circRNA enrichment at the junction level as each gene can host multiple circRNAs. As such, we carried out circRNA enrichment using an over-representation test of the host genes using the *enrichGO* function from clusterProfiler. To detect groups enriched in common BSJs, those included in differential expression testing were compared against all abundant BSJs detected in that cohort (>10 reads in the smallest sample group). To detect categories enriched in differentially expressed BSJs, those that were significant were compared to all BSJs included in differential expression testing. For all enrichment analyses, where possible, categories that passed the multiple testing threshold in the PPMI (FDR < 0.05) were validated in the ICICLE-PD cohort.

### Relating RNA expression to previous work

Genes reported to be differentially expressed in PD were taken from previously published blood RNA sequencing studies^70–73^, resulting in a total of 354 genes. GWAS loci associated with PD risk were obtained from the current largest GWAS meta-analysis^10^. Expression of the nearest genes to these loci was then compared with 70 detected in PPMI and 69 detected in ICICLE-PD. Genes that host established pathogenic parkinsonism variants were obtained from the Genomics England Parkinson Disease and Complex Parkinsonism panel v1.111 (accessed 10/22)^145^. Out of 35 genes, 30 were detected at sufficient expression levels in the PPMI and ICICLE-PD cohorts.

### Classification of Parkinson’s disease status using RNA expression

The following features were used for classification: normalised counts (gene, BSJ and FSJ) counts after Variance Stabilising Transformation as implemented in DESeq2^134^. Circular to linear (BSJ:FSJ) ratios were extracted from CIRIquant^131^. We also explored the use of circRNA differential expression imbalance as a classification feature. The principle was to compare the expression value of each BSJ from a sample we want to classify with the average expression value in controls, subsequently assessing whether the directional deviation from the mean (i.e., over- or under-expression) was consistent with the direction observed in the training group (PPMI). Each BSJ was scored as 1 when the directions agreed or as 0 when the directions disagreed. BSJ scores were summed per sample and the ability of this sum to discriminate between cases and controls was assessed.

For gene predictors, we selected those that were nominally significantly differentially expressed (*P*-value < 0.05). For BSJ, FSJ, BSJ:FSJ ratio and BSJ imbalance models, we used all highly expressed junctions. We investigated the following sets of features: Expression levels by gene (Gene), BSJ counts (BSJ), circular to linear ratios (BSJ:FSJ ratio), circular RNA imbalance (BSJ imbalance), expression levels by gene and BSJ counts (Gene and BSJ). With the exception of the BSJ imbalance (see above), the ability of the selected sets of features to classify cases and controls was assessed using regularised logistic regression as implemented in *glmnet* v4.1.6^146^ and quantified using the area under the receiver operating characteristic curve (AUC). AUCs and their 95% confidence intervals (Delong’s method) were calculated using pROC v1.18.0^147^. Classifiers were derived from the PPMI cohort, using the *nestcv.glmnet* function from *nestedcv* v0.4.4^148^, and 10 fold cross-validation for both inner and outer cross-validation steps. Final model parameters were derived from a cross-validation fold fit on PPMI data. Model generalisability was assessed in the ICICLE-PD cohort.

### Statistical analyses

All statistical analyses were carried out in R v4.2.1. Correlations were assessed by Spearman’s rank correlation (*cor.test*), reported in the text as Spearman’s rho (ρ). We use the term imbalance to describe whether there is an excess of loci (genes or junctions depending on the context) that are overexpressed in PD compared to controls. It describes the number of features showing log_2_ fold change >0.1 divided by the number of loci with log_2_ fold change >0.1 or <−0.1. Significance was assessed using a two-sided exact binomial test (*binom.test*). Group differences were assessed using a Wilcoxon rank-sum test (*wilcox.test*). Where appropriate, multiple testing correction was performed using Benjamini-Hochberg or Bonferroni corrections (*p.adjust*).

### Reporting summary

Further information on research design is available in the Nature Research Reporting Summary linked to this article.

### Supplementary information

Supplementary Materials

Reporting Summary

Supplmentary Data 1

Supplmentary Data 2

Supplmentary Data 3

Supplmentary Data 4

Supplmentary Data 5

Supplmentary Data 6

Supplmentary Data 7

Supplmentary Data 8

Supplmentary Data 9

Supplmentary Data 10

Supplmentary Data 11

## Data Availability

PPMI raw RNA sequencing and corresponding clinical data are available from https://www.ppmi-info.org/. Due to Ethics, ICICLE-PD raw RNA sequence data is stored locally (https://data.ncl.ac.uk/), but is available for non-commercial, collaborative use upon reasonable request. Summary data used to generate summary statistics and figures are included as supplementary datasets. Data are available under the terms of the Creative Commons Attribution 4.0 International license (CC-BY 4.0).

## Code availability

The code used in this analysis is available at https://github.com/bj-w/PD-lin-circ-RNA-paper.
