# Supplementary material for: Early-stage idiopathic Parkinson’s disease is associated with reduced circular RNA expression
Source: NPJ Parkinsons Dis. 2024 Jan 20;10:25. doi: 10.1038/s41531-024-00636-y (PMC10799891; doi:10.1038/s41531-024-00636-y)
Supplement: Supplementary file 1 — Supplementary Materials [file 41531_2024_636_MOESM1_ESM.pdf]

# Early-stage idiopathic Parkinson's disease is associated with reduced circular RNA expression.

## Supplementary Figures:

**Supplementary Figure 1. Sequencing depths of each cohort.**

**Supplementary Figure 2. Computational detection of circular RNAs.**

**Supplementary Figure 3. Validating clinically reported sex.**

**Supplementary Figure 4. Technical sources of variation when quantifying gene expression.**

**Supplementary Figure 5. Technical sources of variation when quantifying circular RNA expression.**

**Supplementary Figure 6. Contribution of covariates to gene- and circRNA-specific expression.**

**Supplementary Figure 7. RNA differential expression.**

**Supplementary Figure 8. Expression levels of linear RNAs and genes do not differ between study groups.**

## Supplementary Tables:

**Supplementary Table 1. Participant characteristics**

**Supplementary Table 2. Classification strategy summaries (classification of early-stage idiopathic Parkinson's disease status)**

**Supplementary Figure 1**

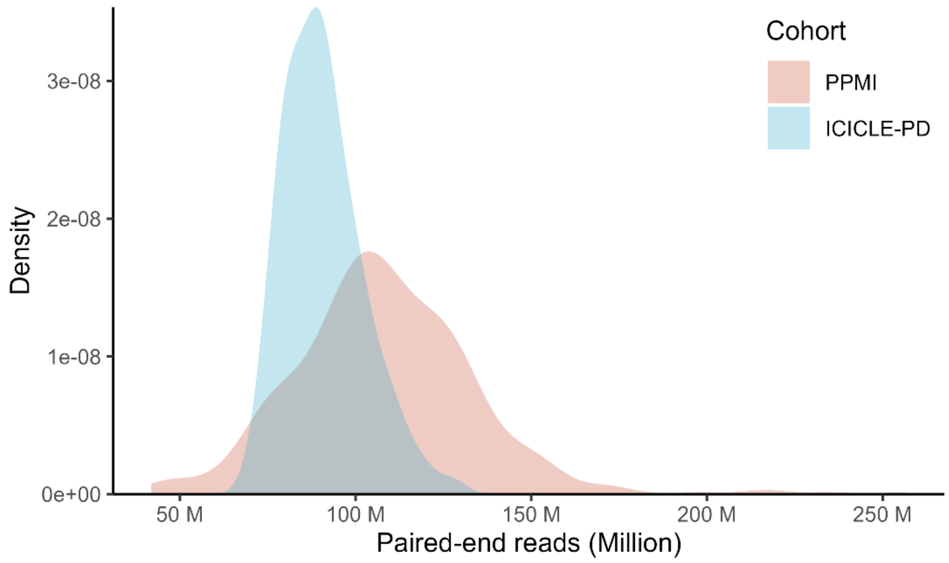


**Supplementary Figure 1. Sequencing depths of each cohort.**
Density plots showing the distributions of sequencing depths (based on the number of paired-end reads sequenced in each sample, paired-end reads per million reads) in PPMI and ICICLE-PD cohorts.

## Supplementary Figure 2

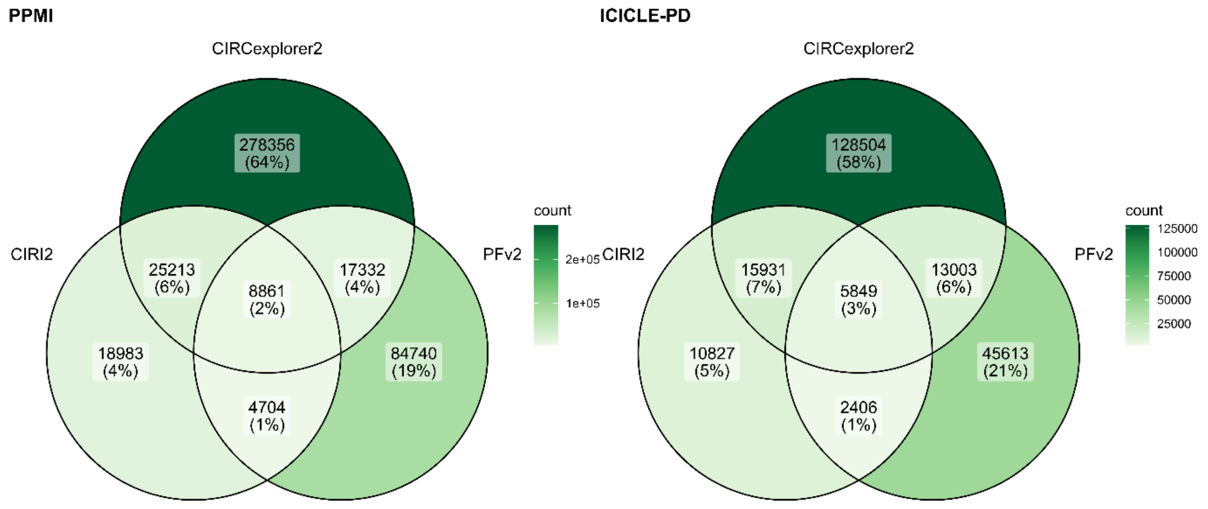


**Supplementary Figure 2. Computational detection of circular RNAs.**
Venn diagrams showing the number of circRNAs (defined as a back-spliced junction or BSJ) detected in PPMI and ICICLE-PD cohorts. The Venn diagram shows the overlap of BSJs detected by CIRI2, CIRCexplorer2 and PTESfinder v2 (PFv2). The proportion of the total BSJs detected in each cohort is given in brackets.

**Supplementary Figure 3**

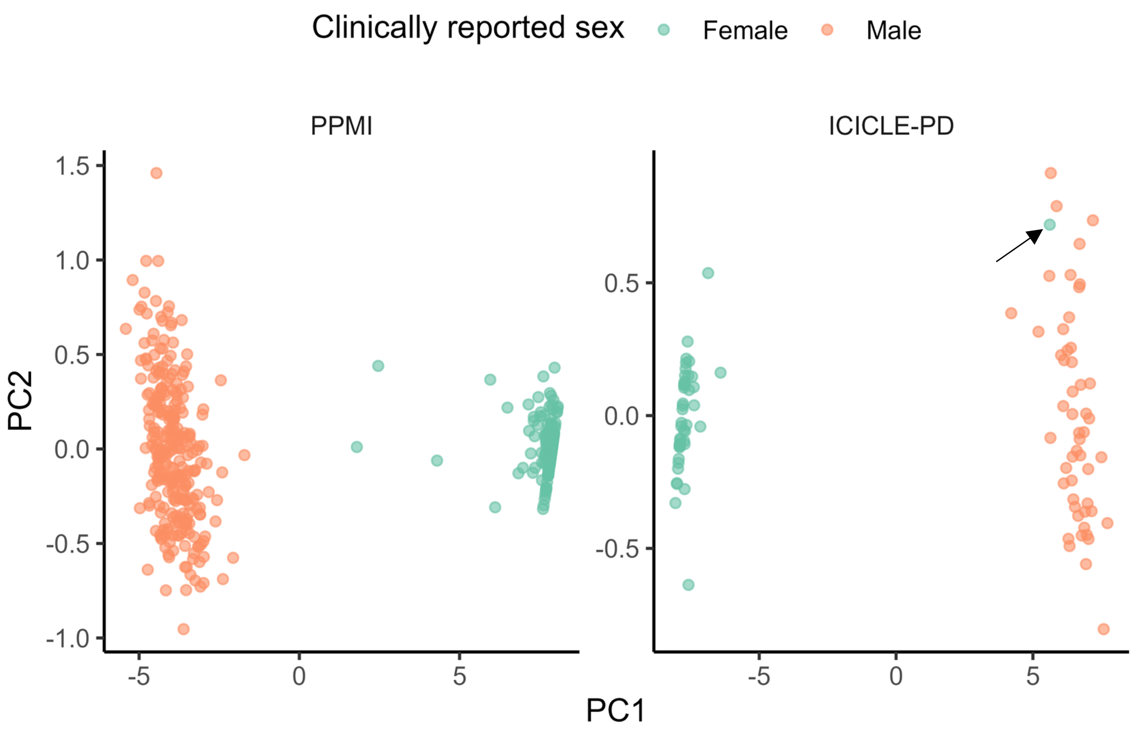


**Supplementary Figure 3. Validating clinically reported sex.**
Scatter plot showing the first two principal components of variance-stabilised transformed expression of selected Y-chromosome genes (*RPS4Y1*, *KDM5D*, *DDX3Y* and *USP9Y*). This identified one sample in the ICICLE-PD cohort (arrow) with a mismatch between clinically reported sex and sex-related gene expression. The discrepancy was checked with clinical data, confirmed to be an error, and changed from female to male.

**Supplementary Figure 4**

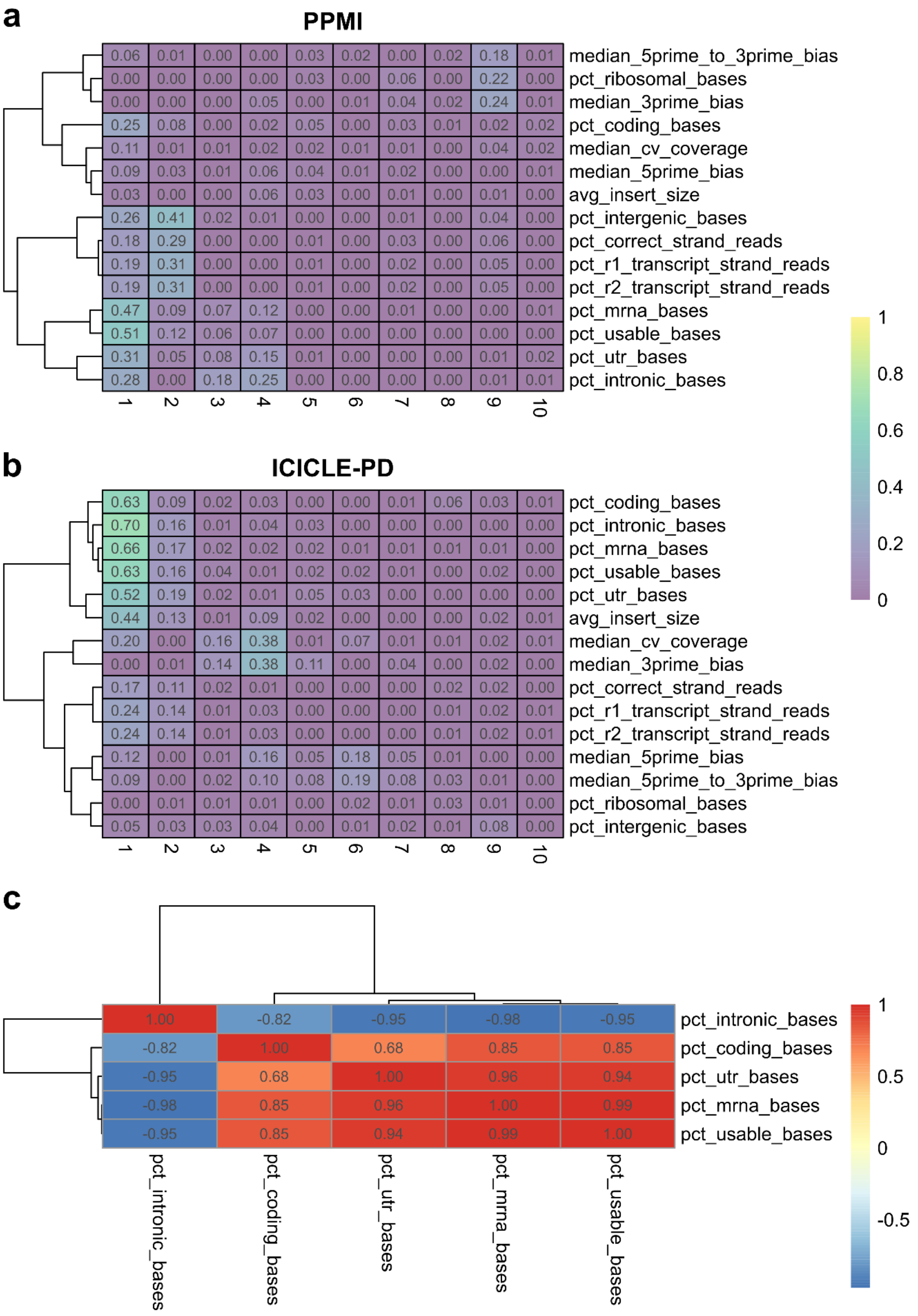


**Supplementary Figure 4. Technical sources of variation when quantifying gene expression.**
Principal components of gene expression were generated for each cohort. Technical metrics were then regressed against the first ten principal components with the $R^2$ reported. **a)** In the PPMI, the percentage of usable bases was identified as explaining a large amount of PC1 ($R^2$>0.5, FDR < 0.05). **b)** In ICICLE-PD, several metrics explained a large proportion of PC1 ($R^2$>0.5, FDR < 0.05). These were tested for collinearity (Spearman's rho >0.9) in **c)** with percentage of intronic reads and percentage of coding bases suitably uncorrelated.

**Supplementary Figure 5**

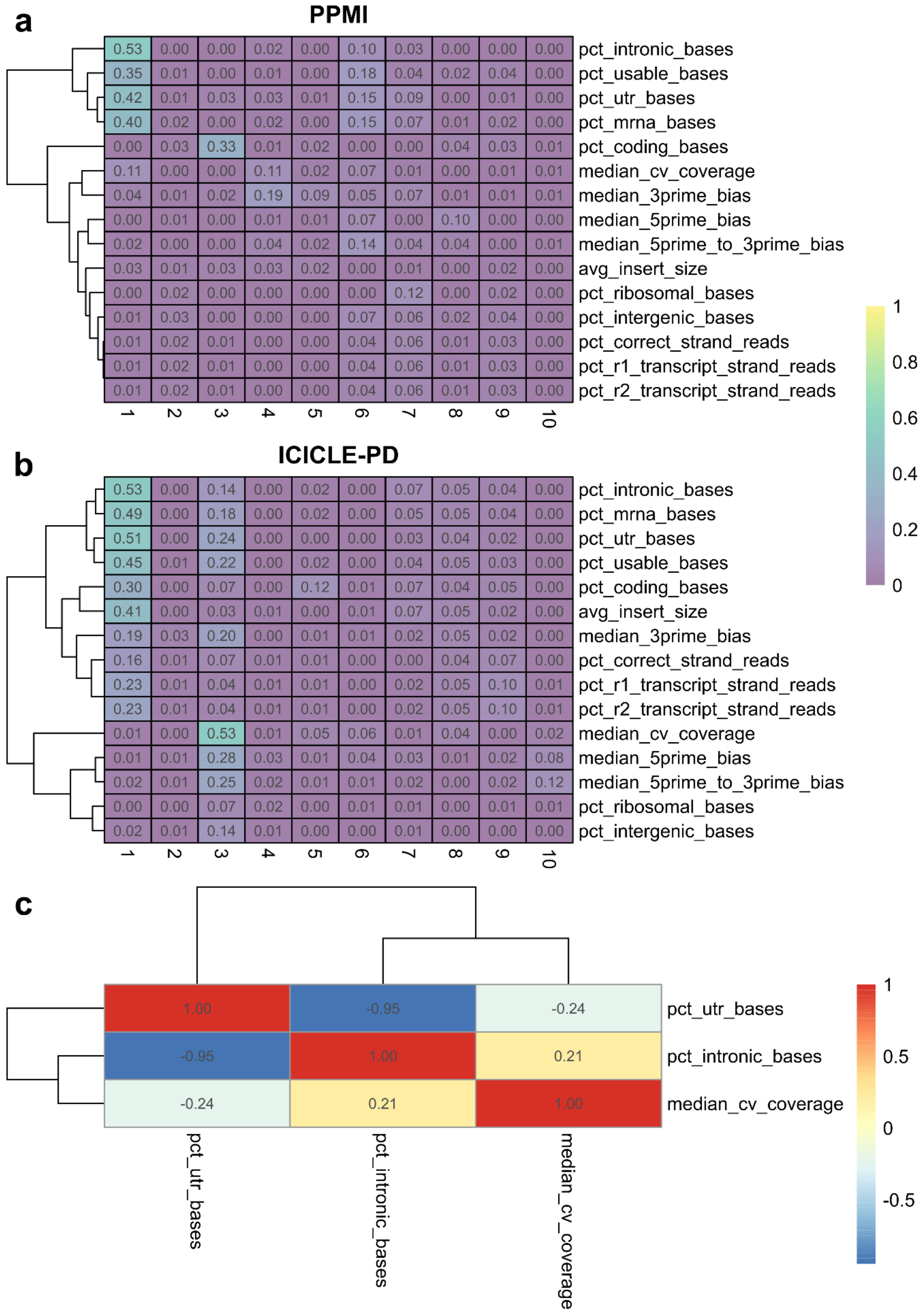


**Supplementary Figure 5. Technical sources of variation when quantifying circular RNA expression.**
Principal components of circular RNA expression were generated for each cohort. Technical metrics were then regressed against the first ten principal components with the $R^2$ reported. (**a**) In PPMI, percentage of intronic bases was identified as explaining a large proportion of PC1 ($R^2$ >0.5, FDR<0.05). (**b**) In ICICLE-PD, percentage of intronic bases, percentage of UTR (untranslated region) bases and the median coefficient of variance of coverage explained large proportions of PC1 and PC3 (R2>0.5, FDR<0.05). (**c**) When tested for collinearity, percentage of UTR bases was shown to be colinear with percentage of intronic bases (Spearman's rho >0.9).

## Supplementary Figure 6

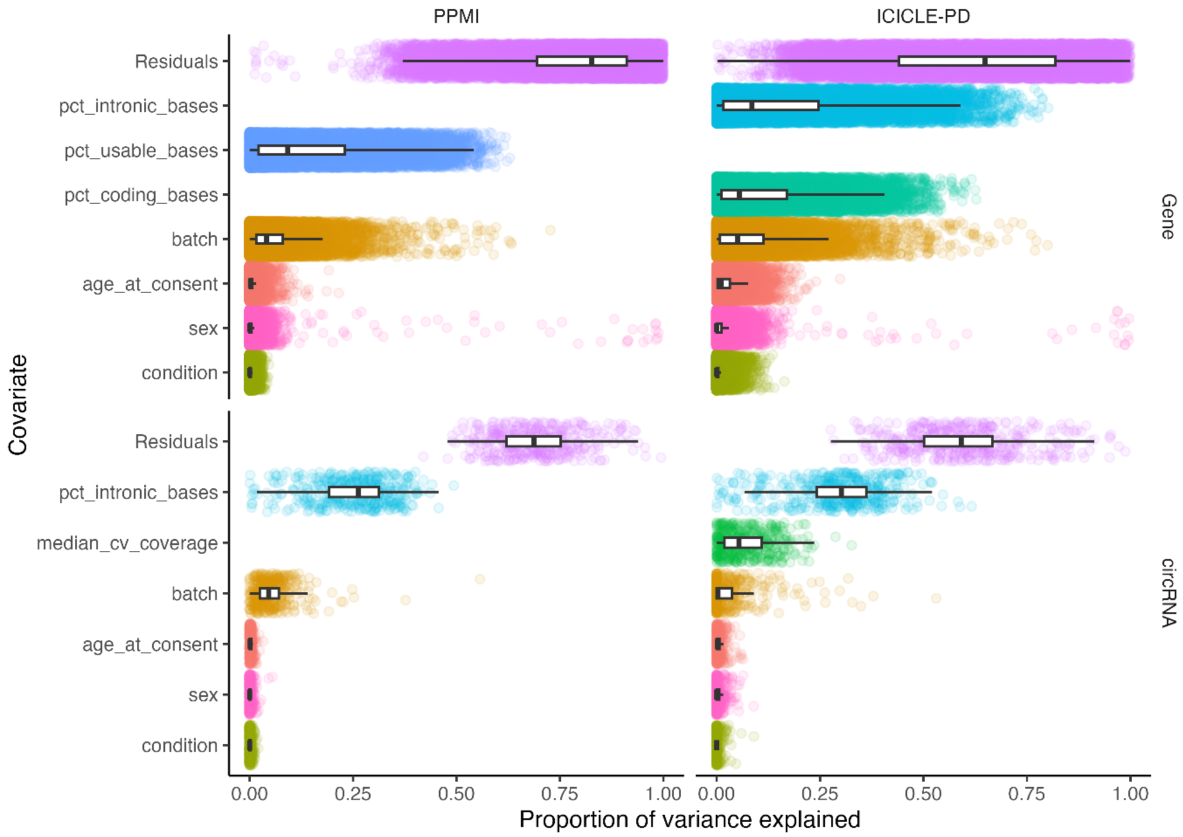


**Supplementary Figure 6**. **Contribution of covariates to gene- and circRNA-specific expression**.

A linear mixed model was fit predicting the expression of each individual gene and circRNA tested. In each model, we included age at collection, sex and sequencing batch, along with RNA and cohort specific technical covariates identified in **Supplementary Figures 4=5**.

**Supplementary Figure 7**

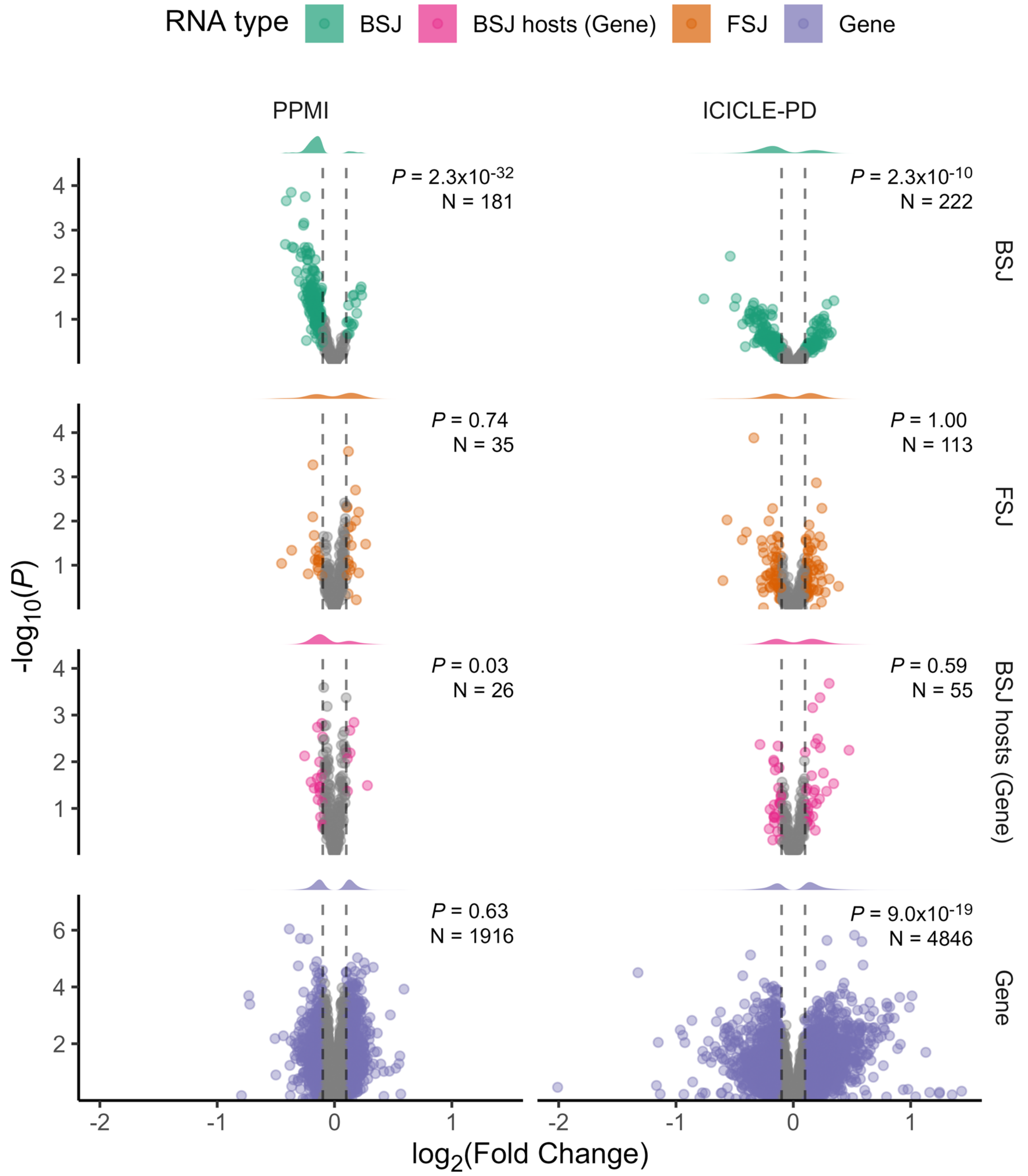


**Supplementary Figure 7**. **RNA differential expression**.
Volcano plots showing the differential expression of RNA types between PD and controls in PPMI and ICICLE-PD. Differential expression was carried out on back-spliced junctions (BSJs), forward-spliced junctions (FSJ), expression of genes which host an abundant BSJ (BSJ hosts) and expression of all genes (Gene). Coloured points indicate RNAs above the fold change threshold (log2 fold change >0.1 or <-0.1, indicated by the dashed grey lines). The imbalance in RNA expression is based on the proportion of RNAs above or below the fold change thresholds (given as N), with significance assessed using a two-sided exact binomial test (given as *P*). Density plots show the distribution of RNAs above or below the fold change thresholds.

## Supplementary Figure 8

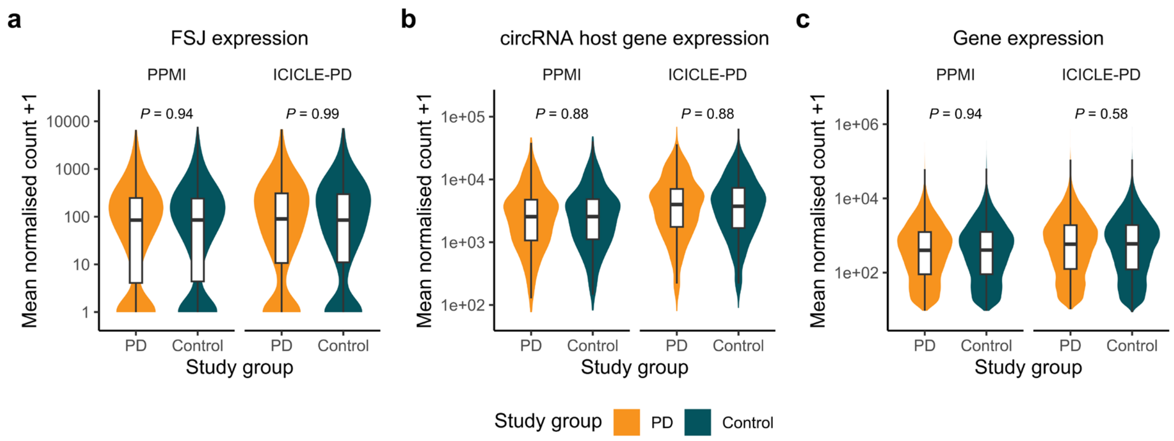


**Supplementary Figure 8. Expression levels of linear RNAs and genes do not differ between study groups**.
Comparing the expression of all BSJs (**a**), genes that host BSJs (**b**) and all genes (**c**) included in differential expression testing between PD and controls in PPMI and ICICLE-PD. *P*-values are derived from a Wilcoxon rank-sum test comparing BSJ expression between PD and controls.

**Supplementary Table 1. Participant characteristics.**

Table showing relevant demographic and clinical characteristics of individuals involved in analysis following sample QC. Where possible, the mean value for each group is given along with the standard deviation (SD). Differences in the proportion of Males/Females between PD and Controls was assessed using a two-sided Fisher's exact test. All other group differences were assessed using a two-sided two-sample T test. MoCa = Montreal Cognitive Assessment. UPDRS-III = 3rd segment of the Unified Parkinson's Disease Rating Scale. NA indicates not available.

| | ***PPMI*** | | | | ***ICICLE-PD*** | | |
|---|---|---|---|---|---|---|---|
| *Variable* | *PD (n = 259)* | *Controls (n = 161)* | *P* | | *PD (n = 48)* | *Controls (n = 48)* | *P* |
| Sex (M/F) | 172/87 | 105/56 | 0.83 | | 29/19 | 23/25 | 3.10E-01 |
| Age at sample collection (years) | 62.9, SD = 9.44 | 60.8, SD = 11.7 | 0.058 | | 64.8, SD = 10.5 | 67.9, SD = 7.36 | 9.40E-02 |
| Age at diagnosis | 62.1, SD = 9.43 | N/A | N/A | | 64.3, SD = 10.5 | N/A | N/A |
| Disease duration (Months) | 4.54, SD = 2.90 | N/A | N/A | | 5.04, SD = 3.41 | N/A | N/A |
| UPDRS-III | 20.2, SD = 8.26 | 1.26, SD = 2.25 | <2.2E-16 | | 23.5, SD = 11.8 | N/A | N/A |
| MoCa | 27.2, SD = 2.39 | 28.3, SD = 1.11 | 5.10E-09 | | 26.0, SD = 3.24 | 27.1, SD = 2.57 | 9.60E-02 |
| Dopaminergic treatment (Y/N) | 0/259 | N/A | N/A | | 44/4 | N/A | N/A |

**Supplementary Table 2. Classification strategy summaries (classification of early-stage idiopathic Parkinson's disease status)**

Table showing summary statistics of each classification strategy. Each strategy was used to classify individuals as belonging to the early-stage idiopathic Parkinson's disease group or the control group. As predictors we used gene expression (Gene), circular RNA expression (back-spliced junctions, BSJ), combined gene and circular RNA expression (Gene + BSJ), linear RNA expression (forward-spliced junctions, FSJ), circular to linear ratio (BSJ:FSJ), and the circular RNA expression imbalance in each individual (BSJ imbalance). For each strategy we give the name, number of features included in the final model, Area under the ROC curve (AUC) and 95% confidence intervals in PPMI and the AUC with 95% confidence intervals in ICICLE-PD.

| Strategy | Number of features | PPMI AUC (95% CI) | ICICLE-PD AUC (95% CI) |
|---|---|---|---|
| Gene | 805 | 0.84 (0.81, 0.88) | 0.59 (0.48, 0.71) |
| BSJ | 15 | 0.61 (0.55, 0.66) | 0.59 (0.48, 0.71) |
| Gene + BSJ | 892 | 0.85 (0.81, 0.89) | 0.60 (0.48, 0.71) |
| FSJ | 15 | 0.57 (0.51, 0.63) | 0.57 (0.46, 0.69) |
| BSJ:FSJ | 21 | 0.58 (0.63, 0.52) | 0.63 (0.51, 0.74) |
| BSJ imbalance | 403 | 0.59 (0.54, 0.65) | 0.59 (0.48, 0.71) |
