# Supplementary material for: Early-stage idiopathic Parkinson’s disease is associated with reduced circular RNA expression
Source: NPJ Parkinsons Dis. 2024 Jan 20;10:25. doi: 10.1038/s41531-024-00636-y (PMC10799891; doi:10.1038/s41531-024-00636-y)
Supplement: Supplementary file 2 — Reporting Summary [file 41531_2024_636_MOESM2_ESM.pdf]

nature portfolio

NPJPARKD-02150R

Corresponding author(s): Gavin Hudson

Last updated by author(s): 1/12/2023

Reporting Summary

Nature Portfolio wishes to improve the reproducibility of the work that we publish. This form provides structure for consistency and transparency in reporting. For further information on Nature Portfolio policies, see our Editorial Policies and the Editorial Policy Checklist.

Statistics

For all statistical analyses, confirm that the following items are present in the figure legend, table legend, main text, or Methods section.

n/a	Confirmed	
☐	☒	The exact sample size (n) for each experimental group/condition, given as a discrete number and unit of measurement
☐	☒	A statement on whether measurements were taken from distinct samples or whether the same sample was measured repeatedly
☐	☒	The statistical test(s) used AND whether they are one- or two-sided<br>*Only common tests should be described solely by name; describe more complex techniques in the Methods section.*
☐	☒	A description of all covariates tested
☐	☒	A description of any assumptions or corrections, such as tests of normality and adjustment for multiple comparisons
☐	☒	A full description of the statistical parameters including central tendency (e.g. means) or other basic estimates (e.g. regression coefficient) AND variation (e.g. standard deviation) or associated estimates of uncertainty (e.g. confidence intervals)
☐	☒	For null hypothesis testing, the test statistic (e.g. F, t, r) with confidence intervals, effect sizes, degrees of freedom and P value noted<br>*Give P values as exact values whenever suitable.*
☒	☐	For Bayesian analysis, information on the choice of priors and Markov chain Monte Carlo settings
☒	☐	For hierarchical and complex designs, identification of the appropriate level for tests and full reporting of outcomes
☐	☒	Estimates of effect sizes (e.g. Cohen's d, Pearson's r), indicating how they were calculated

Our web collection on statistics for biologists contains articles on many of the points above.

Software and code

Policy information about availability of computer code

Data collection	We utilised samples with corresponding demographic (e.g., age at sample collection, sex) and clinical data (e.g., dopaminergic treatment status, disease duration) from two large cohorts of patients with Parkinson's disease (PD) and controls of similar ages and sex. The discovery cohort was obtained from The Michael J Fox Foundation Parkinson's Progression Markers Initiative66 (PPMI, https://www.ppmi-info.org/), while The Incidence of Cognitive Impairment in Cohorts with Longitudinal Evaluation-PD67 (ICICLE-PD, https://www.bam-ncl.co.uk/iciclepd) was used for replication. To study transcriptomic changes in the early stages of PD and to ensure parity between discovery and replication cohorts, only PD patients recently diagnosed with PD (<13 months) were included. PD patients harbouring causative variants in select genes (e.g., LRRK2, GBA, SNCA, PINK1, PRKN) were excluded and are thus all PD cases are idiopathic. Both studies were conducted in accordance with the Declaration of Helsinki and Good Clinical Practice guidelines after approval of local ethics committees of the participating sites66,67.
Data analysis	All statistical analyses were carried out in R v4.2.1. Correlations were assessed by Spearman's rank correlation (cor.test), reported in the text as Spearman's rho (ρ). We use the term imbalance to describe whether there is an excess of loci (genes or junctions depending on the context) that are overexpressed in PD compared to controls. It describes the number of features showing log2 fold change >0.1 divided by the number of loci with log2 fold change >0.1 or <-0.1. Significance was assessed using a two-sided exact binomial test (binom.test). Group differences were assessed using a Wilcoxon rank-sum test (wilcox.test). Where appropriate, multiple testing correction was performed using Benjamini-Hochberg or Bonferroni corrections (p.adjust).

For manuscripts utilizing custom algorithms or software that are central to the research but not yet described in published literature, software must be made available to editors and reviewers. We strongly encourage code deposition in a community repository (e.g. GitHub). See the Nature Portfolio guidelines for submitting code & software for further information.

Data

Policy information about availability of data

All manuscripts must include a data availability statement. This statement should provide the following information, where applicable:

- Accession codes, unique identifiers, or web links for publicly available datasets
- A description of any restrictions on data availability
- For clinical datasets or third party data, please ensure that the statement adheres to our policy

PPMI raw RNA sequencing and corresponding clinical data are available from https://www.ppmi-info.org/. ICICLE-PD raw RNA sequence data is freely available upon request. Summary data used to generate summary statistics and figures are included as supplementary datasets. Data are available under the terms of the Creative Commons Attribution 4.0 International license (CC-BY 4.0).

Research involving human participants, their data, or biological material

Policy information about studies with human participants or human data. See also policy information about sex, gender (identity/presentation), and sexual orientation and race, ethnicity and racism.

Reporting on sex and gender	We use the term sex (defined as either male=M or female=F) as a covariate in analysis.
Reporting on race, ethnicity, or other socially relevant groupings	We did not report on race, ethnicity, or other socially relevant groupings.
Population characteristics	We utilised samples with corresponding demographic (e.g., age at sample collection, sex) and clinical data (e.g., dopaminergic treatment status, disease duration) from two large cohorts of patients with Parkinson's disease (PD) and controls of similar ages and sex. The discovery cohort was obtained from The Michael J Fox Foundation Parkinson's Progression Markers Initiative66 (PPMI, https://www.ppmi-info.org/), while The Incidence of Cognitive Impairment in Cohorts with Longitudinal Evaluation-PD67 (ICICLE-PD, https://www.bam-ncl.co.uk/iciclepd) was used for replication.
Recruitment	Sample recruitment is detailed at https://www.ppmi-info.org/ and https://www.bam-ncl.co.uk/iciclepd
Ethics oversight	Ethics oversight is detailed at https://www.ppmi-info.org/ and https://www.bam-ncl.co.uk/iciclepd

Note that full information on the approval of the study protocol must also be provided in the manuscript.

Field-specific reporting

Please select the one below that is the best fit for your research. If you are not sure, read the appropriate sections before making your selection.

☒ Life sciences ☐ Behavioural & social sciences ☐ Ecological, evolutionary & environmental sciences

For a reference copy of the document with all sections, see nature.com/documents/nr-reporting-summary-flat.pdf

Life sciences study design

All studies must disclose on these points even when the disclosure is negative.

Sample size	We utilised samples with corresponding demographic (e.g., age at sample collection, sex) and clinical data (e.g., dopaminergic treatment status, disease duration) from two large cohorts of patients with Parkinson's disease (PD) and controls of similar ages and sex. The discovery cohort was obtained from The Michael J Fox Foundation Parkinson's Progression Markers Initiative66 (PPMI, https://www.ppmi-info.org/), while The Incidence of Cognitive Impairment in Cohorts with Longitudinal Evaluation-PD67 (ICICLE-PD, https://www.bam-ncl.co.uk/iciclepd) was used for replication.
Data exclusions	As detailed in the manuscript:To study transcriptomic changes in the early stages of PD and to ensure parity between discovery and replication cohorts, only PD patients recently diagnosed with PD (<13 months) were included. PD patients harbouring causative variants in select genes (e.g., LRRK2, GBA, SNCA, PINK1, PRKN) were excluded and are thus all PD cases are idiopathic. Both studies were conducted in accordance with the Declaration of Helsinki and Good Clinical Practice guidelines after approval of local ethics committees of the participating sites66,67.<br>As detailed in the manuscript: Sample level quality control was assessed in both cohorts to identify sample failure, RNA contamination and abnormal global transcriptome issues. Fastq Screen v0.14.1132 and FastQC v0.11.7 (https://www.bioinformatics.babraham.ac.uk/projects/fastqc/) were used to assess contamination and obtain general sequencing metrics. Alignment metrics were obtained using CollectRnaSeqMetrics from Picard v2.27.5 (http://broadinstitute.github.io/picard/) and stats from SAMtools v1.6133. Additionally, PPMI samples that had been previously flagged due to QC issues were removed27. Validation of each participant's clinically recorded sex was based on the normalised and variance-stabilising transformed134 expression of the Y chromosomal genes RPS4Y1, KDM5D, DDX3Y and USP9Y. Principal component analysis was carried out and the first two principal components and was used to detect mismatches by visual inspection. This identified one incorrectly coded individual in ICICLE-PD, which was corrected for analysis. After QC, the final PPMI dataset comprised of 259 PD and 161 controls; the ICICLE-PD dataset comprised of 48 PD patients and 48 controls. We observed no significant differences between the age or sex profiles of cases and controls in either dataset.

Replication	As detailed in the manuscript: We limited all our analysis to early-stage idiopathic Parkinson's disease (PD) patients (i.e., diagnosed <13 months and no known predisposing genetic variation) to age and sex-matched controls, using PPMI as a discovery cohort and ICICLE-PD as a replication cohort. Detailed in the manuscript.
Randomization	As detailed in the manuscript: Like previous large-scale transcriptomic studies, we quantified sources of expression variation at both the sample and RNA level27,137,138. At the sample level, we used univariate linear regression to identify technical sequencing metrics (obtained from Picard and SAMtools) that explained a high proportion of the variance (R2 >0.5) associated with the first 10 principal components of gene and circRNA expression (SFigures 4a-b & 5a-b). At the gene level, based on recommendations139, we excluded highly correlated factors (Spearman's ρ >0.9, SFigures 4c & 5c), subsequently quantifying the contribution of this reduced set of covariates to gene and circRNA expression variation using variancePartition v1.28.3 (SFigure 6). This final set of cohort-specific covariates were then included in regression modelling (see Differential expression).
Blinding	Our analysis compared mRNA and circRNA abundances in early-stage idiopathic PD cases to matched controls in two unrelated cohorts. Blinding of data was not appropriate.

Reporting for specific materials, systems and methods

We require information from authors about some types of materials, experimental systems and methods used in many studies. Here, indicate whether each material, system or method listed is relevant to your study. If you are not sure if a list item applies to your research, read the appropriate section before selecting a response.

Materials & experimental systems

n/a	Involved in the study
☒	☐ Antibodies
☒	☐ Eukaryotic cell lines
☒	☐ Palaeontology and archaeology
☒	☐ Animals and other organisms
☒	☐ Clinical data
☒	☐ Dual use research of concern
☒	☐ Plants

Methods

n/a	Involved in the study
☒	☐ ChIP-seq
☒	☐ Flow cytometry
☒	☐ MRI-based neuroimaging

Plants

Seed stocks	NA
Novel plant genotypes	NA
Authentication	NA
